# Pregnancy outcome and follow-up of offspring of donor oocytes recipient from PCOS patients

**DOI:** 10.1186/s12884-022-05114-y

**Published:** 2022-10-19

**Authors:** Yan Jiang, Jing-Chuan Yuan, Ge Song, Xu-Hui Zhang, Sui-Bing Miao, Xiao-Hua Wu

**Affiliations:** The Center for Reproductive Medicine and Infertility, The Fourth Hospital of Shijiazhuang, Shijiazhuang Obstetrics and Gynecology Hospital affiliated to Hebei Medical University, 206 Zhong-Shan-Dong, Shijiazhuang, Hebei 050011 People’s Republic of China

**Keywords:** PCOS, Oocyte donation, Pregnancy outcome, Birth offspring

## Abstract

**Background:**

The use of donated oocytes (DO) for in vitro fertilization(IVF) treatment in patients with infertility is generally recognized, and females with polycystic ovarian syndrome (PCOS) can participate in oocyte donation programs as donor patients. However, the pregnancy outcomes and offspring follow-up in patients with PCOS as the recipients are unclear. This study was to compare the pregnancy outcomes and follow-up of offspring in PCOS and non-PCOS receptor.

**Methods:**

This was a retrospective cohort study of 62 patients undergoing the oocyte reception program were separated into 2 groups: Group I, PCOS oocyte recipients (*n* = 30); Group II, non-PCOS recipients (*n* = 32). Medical records were reviewed, and rates of fertilization, cleavage, high-quality embryos and blastocysts were compared between PCOS and non-PCOS groups. Rates of implantation, pregnancy, ectopic pregnancy, early abortion, multiple pregnancies, and offspring outcomes were calculated using the first single vitrified-warmed blastocyst transfer (SVBT) analysis between the groups.

**Results:**

The average recipient age and body mass index (BMI) of PCOS and non-PCOS patients was (36.3 ± 2.6 vs. 36.2 ± 2.8, and 23.4 ± 3.9 vs. 23.7 ± 4.0), respectively (*P* > 0.05). The fertilization, cleavage, high-quality embryos and blastocyst rates were not significantly different between the PCOS and non-PCOS groups. Rates of implantation, pregnancy, ectopic pregnancy, early abortion, and multiple pregnancies were not significantly different in SVBT between the PCOS and non-PCOS groups. The incidence of complications, such as pre-eclampsia or gestational diabetes, between PCOS and non-PCOS groups was similar (11.8% vs.11.1%, 5.9% vs.5.5%; *P* > 0.05). Preterm births were also similar (11.8% vs.16.7%, *P* > 0.05). Donor oocytes are more likely to be delivered via cesarean Sect. (80.0% vs. 86.7%: *P* > 0.05). The mean gestational age, birth weight, and height were comparable between the 2 groups during full-term delivery.

**Conclusion:**

There was no difference in the pregnancy outcomes and follow-up of the offspring between the PCOS and non-PCOS groups.

## Introduction

Donor oocytes (DO) enables successful pregnancy in many infertile females [1]. In China, the law allows only the donation of oocytes from patients who receive assisted reproductive technology(ART) [2]. Many women with polycystic ovarian syndrome (PCOS) require controlled ovarian hyperstimulation (COH) and in vitro fertilization (IVF). Usually, more oocytes are retrieved, which allows PCOS patients to participate in DO programs as donor patients [3]. PCOS may increase the risk of adverse perinatal outcomes and the long-term health of the offspring [4]. However, few studies have assessed pregnancy outcomes in patients with PCOS receiving oocytes [5, 6]. Vaz GQ et al. showed that PCOS in donors does not seem to affect pregnancy and implantation rates [7]. However, no study has investigated pregnancy outcomes and follow-up of offspring. Therefore, it is essential to determine whether there is a difference in perinatal and neonatal outcomes due to using oocytes from donors with PCOS.

## Patients and methods

### Patients

**Fig. 1:**
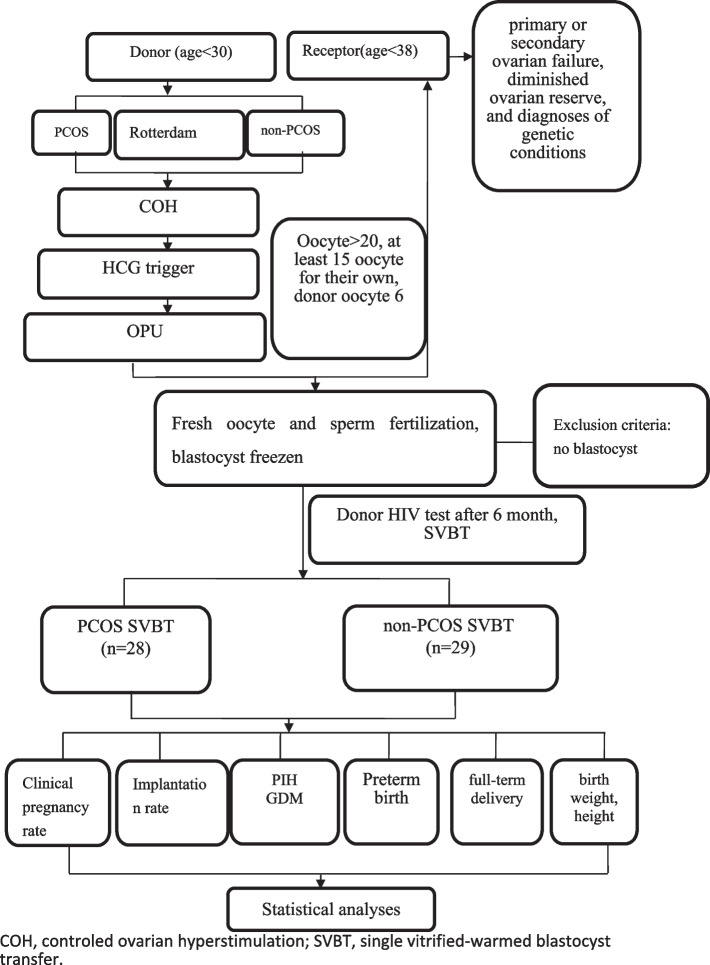
Pregnancy outcome and follow-up of offspring of donor oocytes recipient from PCOS and non-PCOS patients. COH, controled ovarian hyperstimulation; SVBT, single vitrified-warmed blastocyst transfer

This was a retrospective cohort study carried out at the Center for Reproductive Medicine and Infertility, The Fourth Hospital of Shijiazhuang, from March 2015 to May 2020. Sixty-two patients undergoing the oocyte reception program were separated into 2 groups: Group I, PCOS oocyte recipients (*n* = 30); Group II, non-PCOS recipients (*n* = 32). PCOS patients have two or more of the following according to the Rotterdam criteria: (i) amenorrhoea or oligomenorrhea (< 10 menstrual cycles per year), (ii) clinical or biochemical hyperandrogenism, (iii) polycystic morphology on ultrasound, and excluding hyperandrogenemia, such as congenital adrenal hyperplasia, hyperprolactinaemia, or androgen secreting neoplasia [8]. The donor's age both PCOS and non-PCOS patients were < 30 years. The receptor patients included in the analysis were < 38 years at the oocyte reception without physical comorbidities, accepted fresh oocytes and were undergoing their first single vitrified-warmed blastocyst transfer (SVBT). Exclusion criteria donate freezing oocytes, or without single blastocyst transfer (Fig. 1).

Institutional review board approval was obtained; hospital records of deliveries of all patients were reviewed. The Fourth Hospital of Shijiazhuang Ethics Committee approved this study(approval no. 20200004).

### Stimulation, oocyte retrieval, fertilization, embryo culture and scoring

A detail of ovarian stimulation and oocyte retrieval has been previously described by Yan Jiang, et al. [9]. Health regulations permit oocyte donation only from IVF patients who have 20 or more mature oocytes retrieved from a single cycle, of which at least 15 must be kept for their own treatment [10]. So donate 6 oocyte every cycle.

Sperm used for either routine IVF insemination or ICSI procedure using a standard method. Insemination were performed after 38 ~ 40 h of trigger. Fertilization was identified by the presence of two pronuclei approximately 16–19 h after insemination or microinjection. “High-quality embryos” should have 7–9 cells on day 3, contain less than 20% fragments, but might be a little uneven in appearance. On day 3 embryos were transferred into G-2 culture medium in group culture (Vitrolife, Sweden). In the morning of D5 or D6, blastocysts were scored by two experienced embryologist using the system of Gardner and Schoolcraft [10].

### Blastocyst vitrification and warming procedures

Embryos derived from donated oocytes must be cryopreserved and cannot be transferred to prospective recipients, until donors have been screened to be free of communicable diseases after 6 months [2, 11]. The procedure was always performed using one blastocyst for each straw. An artificial shrinkage (AS), using a laser pulse was performed before vitrification. The blastocyst was then moved at room temperature (22–25 °C) to Kitazato (Japan) equilibration solution (ES). After 6–8 min, the blastocyst was quickly washed in vitrification solution (VS) for 45–60 s and transferred onto the straw (Kitazato Japan) using a micropipette and immersed vertically into liquid nitrogen [12].

An Kitazato (Japan) Thaw Kit was used for warming. The carrier containing the embryo was removed from the straw and placed quickly into the dish containing the thawing medium (thawing solution) preheated at 37 °C. The blastocysts immediately fell from the device and could be easily identified in the medium. After 1 min, blastocysts were transferred to the DS medium (dilution solution) for 3 min at room temperature 22–25 °C. In the last two step, blastocysts were placed for 5 min, in the WS1 medium and WS2 (washing solution). The embryo was then returned to G-2 medium for culture until transfer. At this stage, an assessment was performed on an inverted microscope to establish if the embryo survived based on morphological integrity of the ICM and trophectoderm. After 1 or 2 h of culture the embryo was reassessed again and often the re-expansion of the blastocoel was reported; this indicated that the embryo physiologically survived the warming procedure. Embryo transfer was normally performed within 2 or 3 h. All programmed warmed cycles, both at D5 and D6, were transferred in D5 endometrium [12].

### Clinical outcome

Observation of the gestational sac and fetal heart by B ultrasound at 35 days after implantation was diagnosed as clinical pregnancy. The implantation rate was defined as the ratio between the number of gestational sacs and fetal heart observed under B ultrasound and the number of transferred blastocysts. Implantation rates, pregnancy rates, and multiple pregnancy rate of SVBT were analyzed.

### Perinatal and neonatal outcomes

Patients in both groups were given the same standard high-risk obstetric care under the care of the same group of obstetricians. Perinatology consultants were involved whenever there were additional high-risk factors such as Pregnancy-induced hypertension (PIH), gestational diabetes (GDM), or preterm birth (PTB; live birth before 37 weeks gestation).

PIH is defined as new onset of hypertension after the 20th week of gestation with or without proteinuria. GDM is defined as a glucose intolerance of varying severity with onset or first recognition during pregnancy [13].

### Data analysis

Statistical analyses were performed using SPSS 19.0 statistical software (SPSS Inc.). The results are presented as the mean ± standard deviation (SD). The mean values of two groups were compared using the independent samples t-test. Percentages were compared using the χ2 test and *P* < 0.05 was considered statistically significant.

## Results

1. PCOS (*n* = 30) and non-PCOS (*n* = 32) patients basic situation. The average recipient age and body mass index (BMI) of PCOS (*n* = 30) and non-PCOS (*n* = 32) patients was 36.3 ± 2.6 vs. 36.2 ± 2.8, 23.4 ± 3.9 vs. 23.7 ± 4.0, respectively (*P* > 0.05). ICSI rate between PCOS and non-PCOS recipients was similar (66.7%(20/30) vs. 62.5%(20/32), *P* > 0.05). The fertilization, cleavage, high-quality embryos and blastocysts rates were not significantly different between the PCOS and non-PCOS groups (Table 1).
2. Clinical pregnancy results in SVBT between the PCOS (*n* = 28) and non-PCOS (*n* = 29) groups (Fig. 1). Rates of clinical pregnancy (67.9% vs. 68.9%, *P* > 0.05), implantation (67.9% vs. 68.9%, *P* > 0.05), ectopic pregnancy (5.3% vs. 5.0%, *P* > 0.05),and early abortion (5.3% vs. 5.0%, *P* > 0.05) were not significantly different in SVBT between the PCOS and non-PCOS groups (Table 2).
3. Obstetrical outcome after oocyte donation and mode of delivery. The incidence of perinatal complications, such as PIH, and gestational diabetes, between PCOS (*n* = 17) and non-PCOS (*n* = 18) groups was similar (11.8% vs.11.1%, 5.9% vs.5.5%; *P* > 0.05). The incidence of preterm birth was similar (11.8% vs.16.7%, *P* > 0.05) (Table 2).
4. Patients who conceived with donor oocytes were more likely to delivered via cesarean section which appears to be associated with a higher rate of non-elective, rather than elective cesarean sections. The cesarean section rate between PCOS (*n* = 15) and non-PCOS (*n* = 15) groups in full-term delivery (FTD) was similar (80.0% vs. 86.7%: *P* = 0.624) (Table 3).
5. Infant outcome after oocyte donation in full-term delivery between the PCOS (*n* = 15) and non-PCOS (*n* = 15) groups. The mean gestational age(38.1 ± 1.2 vs. 38.4 ± 1.3, *P* > 0.05), birth weight(3281 ± 356 vs. 3302 ± 373, *P* > 0.05), height (50 ± 1.2 vs. 50 ± 1.3, *P* > 0.05), and boy ratio (40% vs. 60%, *P* > 0.05) were comparable between the 2 groups during full-term delivery (Table 3).

**Table 1:** Characteristics of patients receiving oocytes from the PCOS and non-PCOS groups

	PCOS recipients (30)	non-PCOS recipients (32)	t/χ2	*P*
Female age^b^	36.3±2.6	36.2±2.8	0.716	0.476
BMI^b^	23.4±3.9	23.7±4.0	0.620	0.537
Donor oocyte	6	6		
ICSI rate^a^	66.7(20/30)	62.5(20/32)	0.117	0.732
Fertilization rate^a^	75.3(125/166)	76.4(136/178)	0.057	0.811
Cleavage rate^a^	98.4(123/125)	97.8(133/136)	0.127	0.721
High-quality embryo rate^a^	43.9(54/123)	42.1(56/133)	0.084	0.772
Blastocysts rate^a^	39.0(48/123)	37.6(50/133)	0.550	0.814

^a^N (%)

^b^Mean (SD)

Fertilization rate: 2PN/MII

**Table 2:** Clinical pregnancy results in SVBT between the PCOS and non-PCOS groups

	PCOS recipients (28)	non-PCOS recipients (29)	χ2	*P*
Survival rate	100	100		
Clinical pregnancy rate^a^	67.9(19/28)	68.9(20/29)	0.008	0.928
Implantation rate^a^	67.9(19/28)	68.9(20/29)	0.008	0.928
ectopic pregnancy^a^	5.3(1/19)	5.0(1/20)	0.001	0.970
early abortion^a^	5.3(1/19)	5.0(1/20)	0.001	0.970
Multiple pregnancy rate	0	0		
Perinatal complication				
PIH^a^	11.8(2/17)	11.1(2/18)	0.004	0.952
GDM^a^	5.9(1/17)	5.5(1/18)	0.002	0.967
Preterm birth^a^	11.8(2/17)	16.7(3/18)	0.172	0.679

^a^N (%)

**Table 3:** Pregnancy outcomes between the PCOS and non-PCOS groups

	PCOS recipients	non-PCOS recipients	*t*/χ2	*P*
FTD, full-term delivery	15	15		
Cesarean section^a^	80.0(12/15)	86.7(13/15)	0.240	0.624
Gestational age^b^	38.1±1.2	38.4±1.3	0.679	0.467
Boy ratio (boy/girl)^a^	40(6/9)	60(9/6)	1.200	0.273
Weight^b^	3281±356	3302±373	1.156	0.371
Height^b^	50.2±1.2	50.1±1.3	0.639	0.346

^a^N (%)

^b^Mean (SD)

## Discussion

### DO pregnancy outcomes

Since the first successful use of donated oocytes in 1984, many couples have used donor oocytes to treat infertility. Donor cycles represent IVF centers’ first comparable performance measure, allowing for internal and external quality control [14].

As a result, there is growing concern about the impact of oocyte donation on maternal and infant outcomes [15]. Some research showed that autologous oocyte and DO recipients had similar rates of pregnancy complications and her offspring with advanced maternal age in IVF pregnancies [16]. However, the results of the meta-analysis indicated that the risk of developing hypertensive disorders in DO pregnancies was significantly higher than that in autologous IVF pregnancies [17].

Patients with DO should be considered as independent risk factors for some adverse perinatal outcomes, mainly hypertensive disorders in pregnancy, preeclampsia, and severe preeclampsia. The reason for obstetric complications in DO pregnancy may involve placental pathology as a result of immunological pathogenesis and hormonal implications [1, 14, 18].

A matched-pair DO and autologous oocyte analysis showed that DO patients prefer deliver by caesarean section, but infant birth weights and gestational age were similar [19]. However, a study in Sweden showed that despite restricted the age, weight and health to recipients, DO infants have unfavorable neonatal outcomes: such as born prematurely and lower mean birthweight in comparison to non-donor infants [20].

PCOS pregnancy outcomes.

PCOS is the most common endocrinopathy among women of reproductive age. And PCOS patients prefer to conception of ART [21]. Moreover, pregnancies in women with PCOS are more often complicated by gestational diabetes, pregnancy-induced hypertension, preeclampsia, premature delivery and long-term health of her offspring, such as hyperandrogenism and insulin resistance [4, 22–26].

### PCOS patients as DO

Only a few studies have evaluated PCOS with DO. Oocytes from donors with PCOS demonstrated similar fertilization, clinical pregnancy, implantation, and miscarriage rates as oocytes from normal-appearing ovaries [5]. DO with polycystic ovarian morphology has equivalent pregnancy rates and does not need to be excluded as a potential donors [6].

Furthermore, because the oocytes of PCOS patients have a detrimental effect of high luteinizing hormone on oocyte quality and PCOS has a high familial prevalence, some researchers may worry about the possible propagation of the condition in the next generation of PCOS DO programs [27].

In conclusion, both DO and PCOS adversely affect obstetric and infant outcomes. However, PCOS DO did not influence fertilization rates, clinical pregnancy or miscarriage. However, no study has investigated PCOS DO pregnancy outcomes and offspring follow-up. It remains unknown if the obstetric and infant outcomes of oocytes from donors with PCOS have a double disadvantage. The results of this study point to that no difference in pregnancy outcomes and offspring follow-up between the PCOS and non-PCOS groups. But because statistical tests in very small samples the error rate may be increased. More investigation following this pattern can be recommended at this point. I am sure that sharing the results with health professionals will improve the care of women with these processes.

The total number of oocytes and zygotes is prognositc of live-birth pregnancy in fresh donor oocytes during in-vitro fertilization cycles [28]. Therefore, we selected 6 fresh oocyte donors. The transfer should be the option of choice in OD cycles to avoid the additional increase in risk from multiplicity and single-embryo [29]. Therefore, we chose SVBT in OD cycles. To avoid the influence of age, the recipients included in the analysis were < 38 years of age at oocyte reception. There was a statistically significant relationship between the donor's age and the cumulative live-birth rate. The cumulative live-birth rate for recipients with donors aged < 30 years was the highest [30]. Therefor we limited the age of oocyte donor < 30 years.

Further research focusing on the etiopathogenesis of PCOS pathologies is needed. The mechanism of PCOS is due to genetic factors of the egg or an abnormal uterine environment. PCOS DO provides a mode of PCOS oocyte isolation from the PCOS uterine environment of hyperandrogenism. Further research is needed to determine the incidence of PCOS in daughters and its recipients of the homologous egg of patients with PCOS.

### Limitations of the study

We included only 62 oocyte donor patients, and 30 were diagnosed with PCOS. This small number of pregnancies and infants can potentially cause errors in statistical analyses. This study only collected the data up to the newborn. More research should focus on long-term health of PCOS DO’s offspring, such as hyperandrogenism and insulin resistance.

## Conclusion

There was no difference in the pregnancy outcomes and follow-up of the offspring between the PCOS and non-PCOS groups.

## Data Availability

The datasets used during the present study are available from the corresponding author upon reasonable request.
